# Virtual avatar communication task eliciting pseudo-social isolation and detecting social isolation using non-verbal signal monitoring in older adults

**DOI:** 10.3389/fpsyg.2025.1507178

**Published:** 2025-03-14

**Authors:** Ayumi Takemoto, Miyuki Iwamoto, Haruto Yaegashi, Shan Yun, Risa Takashima

**Affiliations:** ^1^Institute of Development, Aging and Cancer, Tohoku University, Sendai, Miyagi, Japan; ^2^Bioinformatics Laboratory, Riga Stradins University, Riga, Latvia; ^3^Department of Social System Studies, Doshisha Women's College of Liberal Arts, Kyoto, Japan; ^4^Graduate School of Science and Technology, Kyoto Institute of Technology, Kyoto, Japan; ^5^Faculty of Education, Tohoku University, Sendai, Miyagi, Japan; ^6^Faculty of Health Science, Hokkaido University, Sapporo, Hokkaido, Japan

**Keywords:** older adults, social isolation, virtual avatar interaction, facial recognition system, cyberball task

## Abstract

Social isolation and loneliness are two of the main causes of mental health problems or suicide, not only in younger adults but also in older adults. Thus, identifying an effective method to detect social isolation is important in the field of human-machine interaction. However, to the best of our knowledge, no effective method has been developed to elicit pseudosocial isolation tasks to evaluate social isolation detection systems for older adults. This study has two research aims: 1. To develop a virtual avatar conversation cyberball task to evoke pseudosocial isolation in older adults and, 2. to identify non-verbal indicators that replace social isolation in older adults. To achieve these objectives, 22 older men were recruited as participants. They were asked to communicate with two virtual avatars on a monitor and then to rate the follow-up questions provided to evaluate the level of social isolation and emotions; meanwhile, facial expressions and gaze patterns were recorded by a camera and an eye tracker. In the results, the developed virtual avatar conversation cyberball task successfully induced pseudosocial isolation in older adults, and this social isolation was detected by the intensity of inner/outer eyebrow and eyelid movements and the blink frequency.

## 1 Introduction

Human beings are social animals. We spend most of our time with other people, such as friends, colleagues, and classmates (Batson, 1990). Demographic and environmental factors such as marriage, the presence of children, a higher level of education, and a greater number of siblings are negatively associated with perceptions of social isolation (Distel et al., 2010). Factors positively correlated with loneliness included being a man, having physically symptoms, chronic work or social stress, having few social networks, and lack of a spouse's best friends (Hawkley et al., 2008; Cacioppo et al., 2015). Therefore, social isolation has significant effects on both mental and physical health (Cacioppo et al., 2011) and is a particularly strong risk factor for morbidity and mortality, as are smoking, obesity, sedentary lifestyle, and hypertension (Cacioppo et al., 2011). Furthermore, social isolation is one of the main causes of cognitive decline and increases the risk of Alzheimer's disease (Wilson et al., 2007).

On the other hand, loneliness has been defined as an unpleasant statement experienced when there is a mismatch between the interpersonal relationships that one wishes to have and the ones that one currently perceives as lacking (Peplau and Perlman, 1982). Two types of loneliness are generally reported: emotional and social (Weiss, 1975). Emotional loneliness represents a lack of intimate relationships and is associated with the absence of emotional support (Liu and Rook, 2013; Green et al., 2001). Social loneliness, on the other hand, refers to the lack of available and acceptable social networks and is often associated with social values such as the presence of close ties with friends, companionship, and the size of the social network (Liu and Rook, 2013; Green et al., 2001). While loneliness and social isolation do not equate to each other, social isolation and both emotional- or social-loneliness strongly correlate with each other (Ge et al., 2017; Wolters et al., 2023).

In older adults, loneliness is associated with the onset of depression and other common mental health problems (Mann et al., 2022) and is one of the indicators used to detect depression (Groarke et al., 2021). Loneliness and social isolation frequently co-occur and are all too common in older adults (Hwang et al., 2020), and they can induce psychological problems and even suicide (Conejero et al., 2018). In addition, some scientific research have already reported that long-term social isolation has a negative impact on quality of life (QOL) in older adults. Social isolation was significantly and independently related to health-related QOL even when depression, physical comorbidities, age, gender, living alone, employment status, and type of accommodation are taken into account (Hawton et al., 2011), and its links to the COVID-19 pandemic also had a huge negative impact on the QOL of older adults (Newman-Norlund et al., 2022). Countries with high suicide rates among older adults include the European Union (EU) countries, Canada, the United States, Japan, and Asian countries such as Singapore and Taiwan (Conwell et al., 2011). Older men, in particular, are at elevated risk in these countries (Cheng and Lee, 2000; Fässberg et al., 2012). Furthermore, loneliness induces not only mental issues but also frequent physical falls, cognitive decline, and Alzheimer's disease (Lara et al., 2019). Petersen et al. (2020) summarized that with regard to social isolation/exclusion of older adults, falls can lead to social exclusion (Hajek and König, 2017) or rather be a consequence of social isolation (Pohl et al., 2018); namely, living alone plays a role in the frequency of falls (Zhou et al., 2019), and the risk of falls increased for those living alone (jin Choi et al., 2014). Furthermore, social isolation and loneliness are associated with cognitive decline in both Western and Asian populations (Yu et al., 2021; Lara et al., 2019; Holwerda et al., 2014; Beller and Wagner, 2018; Griffin et al., 2020). In the Western population, loneliness is often a contributing factor to the onset of dementia (Rafnsson et al., 2020), as it increases the risk of cognitive decline in older adults (Boss et al., 2015). In the Asian population, both loneliness and social isolation indicated an association with decreased mental state and episodic memory (Yu et al., 2021). In both Asian and Western populations, a stronger negative relationship between social isolation and cognitive function than between loneliness (Yu et al., 2021; Griffin et al., 2020; Beller and Wagner, 2018).

Loneliness, especially chronic loneliness, is associated with altered parasympathetic function (Roddick and Chen, 2021). Social isolation is associated with both systolic and diastolic blood pressure (Shankar et al., 2011), and an increase in social isolation is positively correlated with systolic and diastolic blood pressure. Furthermore, non-verbal signals, such as facial expressions and gaze patterns, are also one of the strongest indicators used to recognize emotion in younger adults (Takemoto et al., 2023a,b; Turabzadeh et al., 2018; Dagar et al., 2016). In gaze patterns, younger adults with high loneliness are more attentive to social threats linked to social rejection (Bangee and Qualter, 2018). Therefore, systems for loneliness or social isolation detection have been developed using wearable sensors (Site et al., 2022; Donovan and Blazer, 2020) to monitor biometrical data in older adults as well. In particular, a combination of behavioral monitoring and computer science technology, such as deep learning and artificial intelligence, are some of the common methodologies used in the last decade (Prenkaj et al., 2023).

While non-wearable emotion detection systems using eye tracking and cameras are one of the most common technologies in younger adults, there are few reports on emotion detection systems using non-wearable devices in older adults because the facial expressions of older adults do not change much; thus, the facial morphology in an older adult is difficult to recognize (Tanikawa et al., 2019; Fölster et al., 2014).

Currently, some activities, including exercise, physical fitness (Roberts et al., 2017; Langhammer et al., 2018) and group activities, such as volunteering, group meetings/discussions, and participatory workshops (Cattan et al., 2005) have been reported to help prevent social isolation. Physical activities, especially moderate, physical activity combined with multitasking, have a positive effect on the mental side of people's lives, such as memory and attention span (Roberts et al., 2017). In addition, increasing social connections, such as the extent to which older adults are connected to family, friends, and the community, is a promising strategy for reducing suicide rates among the late-aged (Oyama et al., 2008; Lapierre et al., 2011).

The main aim of this study is to detect social isolation in older adults feeling using non-wearable devices such as eye tracking and a web cameras. To achieve this objective, two aims were implemented in this study: 1. To develop a virtual avatar conversation cyberball task to induce pseudosocial isolation in older adults and, 2. To identify non-verbal indicators that replace social isolation in older adults.

The conventional methods used to induce social isolation [e.g., cyberball task (Williams et al., 2000) and visual task (Cacioppo et al., 2009)] are artificial and less natural for evaluating social isolation detection systems. In our perspective of the research, social isolation detection systems will be applied in daily life, such as online communication, for supporting older adults' QOL. Communication is one of the social activities in our daily lives (Bakhurst, 1997), and online communication systems have currently been focused on and developed as the primary support system for the lives of older adults (Katz and Moyer, 2004), it is planned to install a social isolation monitoring system that will be installed in an online communication system in the future. Therefore, an online conversation task that induces pseudosocial isolation will initially be developed. After the development of the conversation task, non-verbal data, such as gaze patterns and facial expressions, will be collected by non-wearable devices in pseudosocial isolation in older adults.

In these results, a virtual avatar communication task based on cyberball tasks (Williams et al., 2000) was developed and confirmed whether it induced social isolation in older adults. Furthermore, the blink frequency and intensity of movements around the eyes, such as eyebrows and lids, can be indicators of social isolation in older adults.

It was hypothesized that social isolation can manifest itself in the intensity of muscle movements around the eyes associated with negative facial expressions such as inner/outer brow and eyelids (Sato et al., 2019). The intensity of these areas would specifically be higher in social isolation.

## 2 Materials and methods

The pre-registration of this research was performed using the Open Science Framework (OSF) (Registration DOI: https://doi.org/10.17605/OSF.IO/JGDNY). These experiments were conducted with participants interacting with two avatars through a monitor under two experimental conditions. Participants performed conversation tasks using these avatars. This section introduces the participants' traits, experimental conditions, surveys, and procedures, and then the details of the implemented analysis follow. This study was approved by the Ethics Committee of the Graduate School of Health Science, Department of Health Science, School of Medicine, Hokkaido University in accordance with the Declaration of Helsinki (Approval Number: 23-94).

### 2.1 Participants

A prior power analysis [G^*^Power ver. 3.1 (Faul et al., 2009)] was conducted to determine the minimum sample size, which indicated that the required sample size was a mere 10 individuals; therefore, 22 Japanese males participated in the experiment. Each participant provided written informed consent before the experiment and received JPY 3000–6000 (~USD 20–40) for participating. Table 1 indicates the participants' information.

**Table 1:** Participants' characteristics.

Number of participants	22
Age range	65–88 (73.18 ± 6.07)
UCLA loneliness scale	17.41 ± 5.57
Social interaction anxiety scale (SIAS)	25.64 ± 12.97
Mini-mental state examination (MMSE)	25–30 (28.27 ± 1.52)

### 2.2 Survey

#### 2.2.1 Japanese version of the UCLA loneliness scale version 3 (UCLA-LS3-J)

The Japanese version of the University of California, Los Angeles (UCLA) Loneliness Scale Version 3 (UCLA-LS3-J) consists of 10 items to measure the level of loneliness (Arimoto and Tadaka, 2019), and each item can be rated from 1 (Never) to 4 (Always). The scale was based on the original UCLA loneliness scale (Russell et al., 1980). The total score runs from 10 to 40. Miura et al. reported studies on older-adult loneliness and social isolation using this scale to measure loneliness (Miura et al., 2023, 2024).

#### 2.2.2 Social interaction anxiety scale (SIAS)

The Social Interaction Anxiety Scale (SIAS) consists of 20 items that measure the level of anxiety toward social interactions (Mattick and Clarke, 1998). It was validated by Kanai (2004) and was used in many types of research about human-human (Maeda, 2023) or human-computer interaction (Suzuki et al., 2021) in social anxiety.

#### 2.2.3 Mini-mental state examination (MMSE)

The Mini-mental state examination (MMSE) comprises 11 items and screens for dementia (Mori, 1985). The total score ranges from 0 to 30. Three categories were established based on the MMSE score: 1. Non-Dementia (≤ 27), 2.Mild-Cognitive Impairment (from 24 to 26), and 3. Dementia (≥23) (Wind et al., 1997).

#### 2.2.4 Needs satisfaction index

The Needs Satisfaction Index is based on a scale originally created by Williams et al. (2000) to evaluate social isolation induced by the Cyberball task. Table 2 indicates the questions in which the participants evaluated their level of social isolation under each experimental condition. Each item was rated from 1 (not at all) to 9 (very much). Furthermore, their mood was assessed with four questions: bad-good, sad-happy, tense-relaxed, and rejected-accepted. Participants rated the scores from 1 to 9.

**Table 2:** Items of Needs Satisfaction Index.

**Category**	**Question**
Belonging	**Q1**. How much do you feel you belong to the group?
Self-Esteem	**Q2**. To what extent do you think the other participants value you as a person?
Intensity of ostracism	**Q3**. To what extent did you feel that you were being ignored or excluded by the other participants? **Q4**. To what extent did you feel that you were noticed or included by the other participants?
Perception of group cohesiveness	**Q5**. How much did you like the other players? **Q6**. How much did the other players like you?
Mood	Bad-good; sad-happy; tense-relaxed; rejected-accepted

### 2.3 Apparatus

Two cartoon avatars were presented on a monitor (DELL, 1920 × 1080 pixels, 34.5 × 19.4 cm) and controlled by a native Japanese member of the research team through a Unity game engine in the same room. The viewing distance was 60 cm. Eye movements were recorded by a Tobii Pro Nano with a 60-Hz refresh rate and were calibrated before each session by the Tobii Pro Eye Tracker Manager. Facial expressions were monitored using an FHD camera (30 fps).

### 2.4 Types of interlocutors of virtual avatars in the experiment

**Figure 1:**
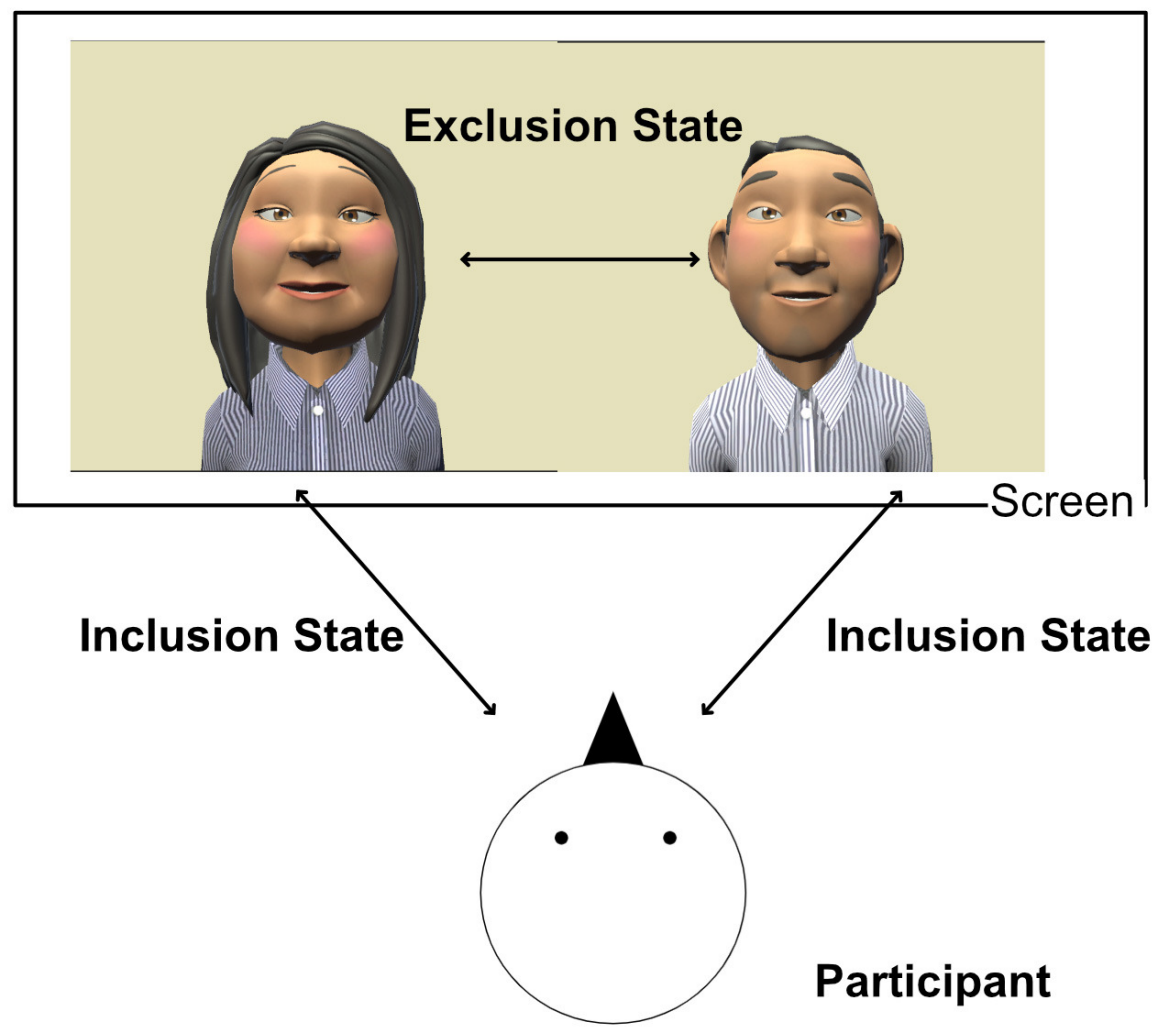
This image indicates the summary of this conversation task. A participant and two avatars communicate together on the screen. The figure was created by Toon people ver 3.1 which is a Unity asset produced by JBGarrazaUnity (2021) and reproduced with permission from the author, licensed under CC BY-NC 4.0.

Two types of animated virtual avatars were prepared as interlocutors in the experiment: male and female avatars. For the determination of a virtual avatar's appearance, several researchers have reported that there is no significant difference between a human-like and an animated avatar in terms of age, gender, and ethnicity of virtual avatars in terms of frustration levels, preference, and the level of rapport (Hone, 2006; Richards et al., 2020; Pratt et al., 2007). Several different types of virtual avatar pictures were created that resembled the general Japanese appearance–light skin tone, brown eyes, and dark hair (Peck et al., 2022; Lin et al., 2016), current casual clothing, hairstyle–and then students in the Department of Medicine, Tohoku University were asked to rate each avatar's impression using the 5-point Likert scale (e.g., 1. Friendly, 5. Unfriendly) based on the questionnaire created by Pratt et al. (2007), and two virtual avatars for each gender with a middle score for the impression were selected (Figure 1).

### 2.5 Experimental procedure

The interaction of the conversation cyberball task involved roughly structured dialogues between the participant and two virtual avatar interlocutors (Figure 1). Each session consisted of 30 trials. The participant and the avatar interlocutors communicated together, and the avatar interlocutors led the conversation with a closed-ended question (participants can answer “yes” or “no”) based on the topic. The conversation task consisted of two states: exclusion and inclusion. In the excluded state, only avatar interlocutors were communicating but without a participant. On the other hand, in the inclusion state, the participant was included in the conversation; therefore, either avatar interlocutor was asking a question of the participant. A member of the research team was in the same room to control the experimental system based on the participants' reactions. Participants were offered breaks between sessions. Each participant underwent a total of two sessions: isolation and company conditions. In the company condition, participants were involved in eighteen to thirty (60%) inclusion conditions. On the other hand, in the isolation condition, participants were involved in 4 out of 30 (13%) inclusion conditions. Conversation topics were employed in seasons and daily life where it was already confirmed that they did not have a negative effect on emotion (Takemoto et al., 2023b). The combination of conversation topics and experimental conditions was assigned randomly to participants. Before starting the main session, participants practiced talking to the virtual avatar about animals in nine trials. Both conditions were set up so that the two avatars were already acquainted, but the participants met them for the first time. In order to evaluate the effect of the experimental condition, the participants filled out in a Needs Satisfaction Index after each session.

### 2.6 Analysis

#### 2.6.1 Eye tracking

Two types of gaze features were computed based on the recorded eye positions of the participants' right and left eyes: the number of saccades and fixation duration. All data analyses were conducted using MATLAB (MathWorks, Natick, MA) and Python. Fixation points were obtained using the EyeMMV toolbox (Krassanakis et al., 2014). In this system, a two-step spatial dispersion threshold was used for fixation identification. First, in cases where the length between the mean point and the record was greater than the first allowed value (set at 2 degrees in this study), the mean horizontal and vertical coordinates were computed, and if the distance was greater than 2°, new solid clusters were generated. The distance between the mean point and each record in each cluster was then calculated, and if the distance for a record was greater than 1°, the record was not used as a fixation. In this study, the minimum fixation duration was 100 ms.

The number of saccade occurrences in each session was used as an indicator used to detect the emotions. Specifically, the number of saccades occurring per second was computed and used as the saccade frequency value. The average duration of each gaze fixation viewpoint in each session was also computed and used as the value of fixation duration.

#### 2.6.2 Facial expression

Using OpenFace (Baltrusaitis et al., 2016), a facial imaging process tool, was used to extract facial features related to each participant's Action Units (Ekman and Rosenberg, 1997), using the video data. In this study, the analysis was limited to the Facial Action Units (FAUs) around the eyes (e.g., AU1, AU2, AU5, and blink frequency) because participants moved their mouths; thus, FAUs around the mouth were associated with conversation. Because FAUs around the eyes often reflect subtle emotional and attentional changes, data points with FAU values of zero were excluded to efficiently detect such changes, and then the average values of FAUs was computed. Corresponding t-tests were also used to detect statistically significant differences between experimental conditions.

## 3 Results

A *post hoc* analysis was conducted using G*Power ver. 3.1 (Faul et al., 2009) to confirm sufficient statistical power (Power = 0.99). The characteristics of the participants are shown in Table 1. This section reports the results of the survey, eye movements, and facial expressions under each experimental condition. The collected data revealed that the isolation condition successfully elicited fewer favorable impressions and positive emotions than the company condition. Furthermore, there are significant differences between the isolation and company conditions in facial expressions.

### 3.1 Effect of social isolation on emotion

**Figure 2:**
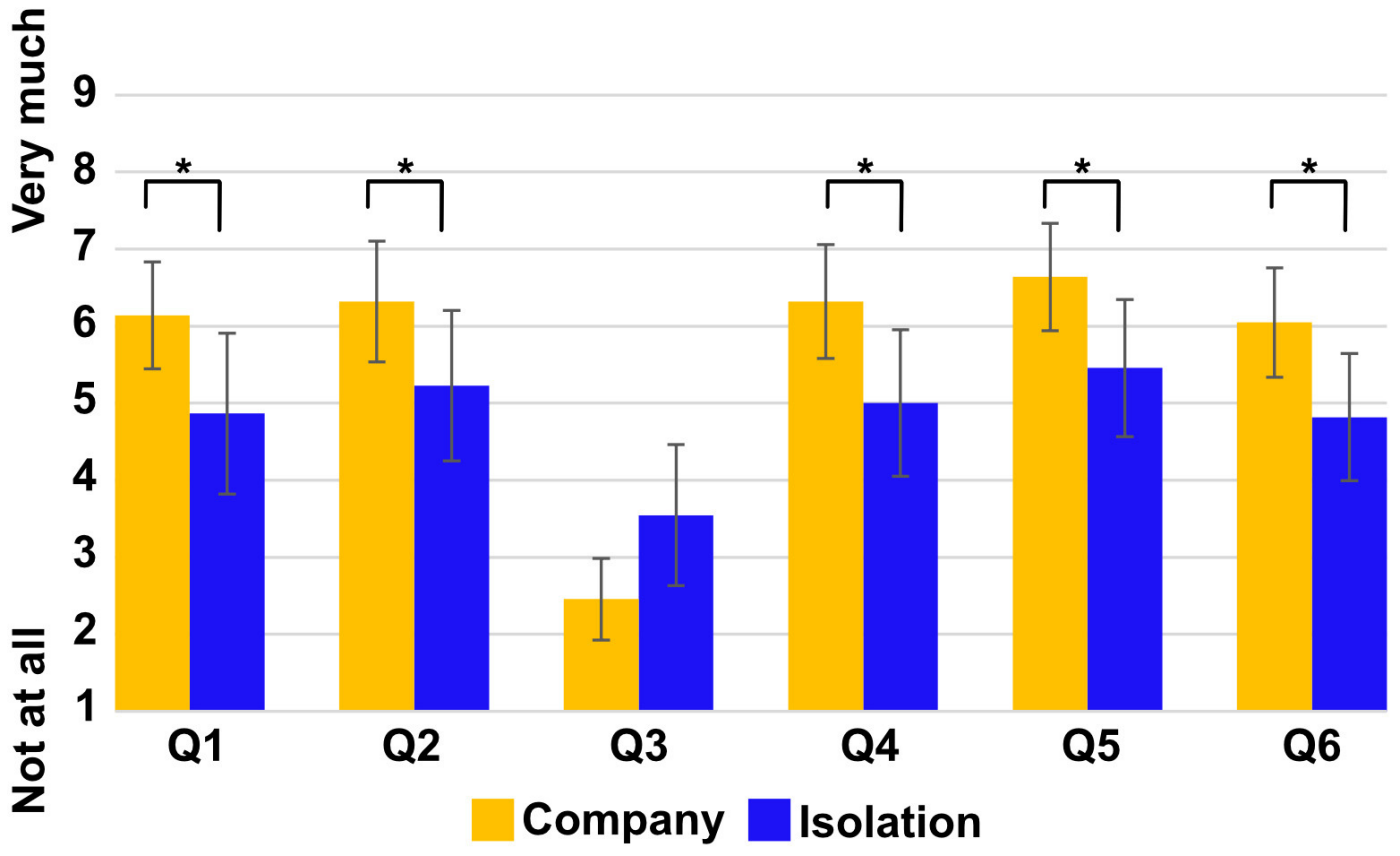
Average score in each question in the Needs Satisfaction Index between the company (yellow boxes) and isolation (blue boxes) conditions. The error bars indicate 95% confidence interval (CI). Q3 is a revised question. *Indicates that the p-value was less than 0.008, which is statistically significant after the Bonferroni correction.

**Figure 3:**
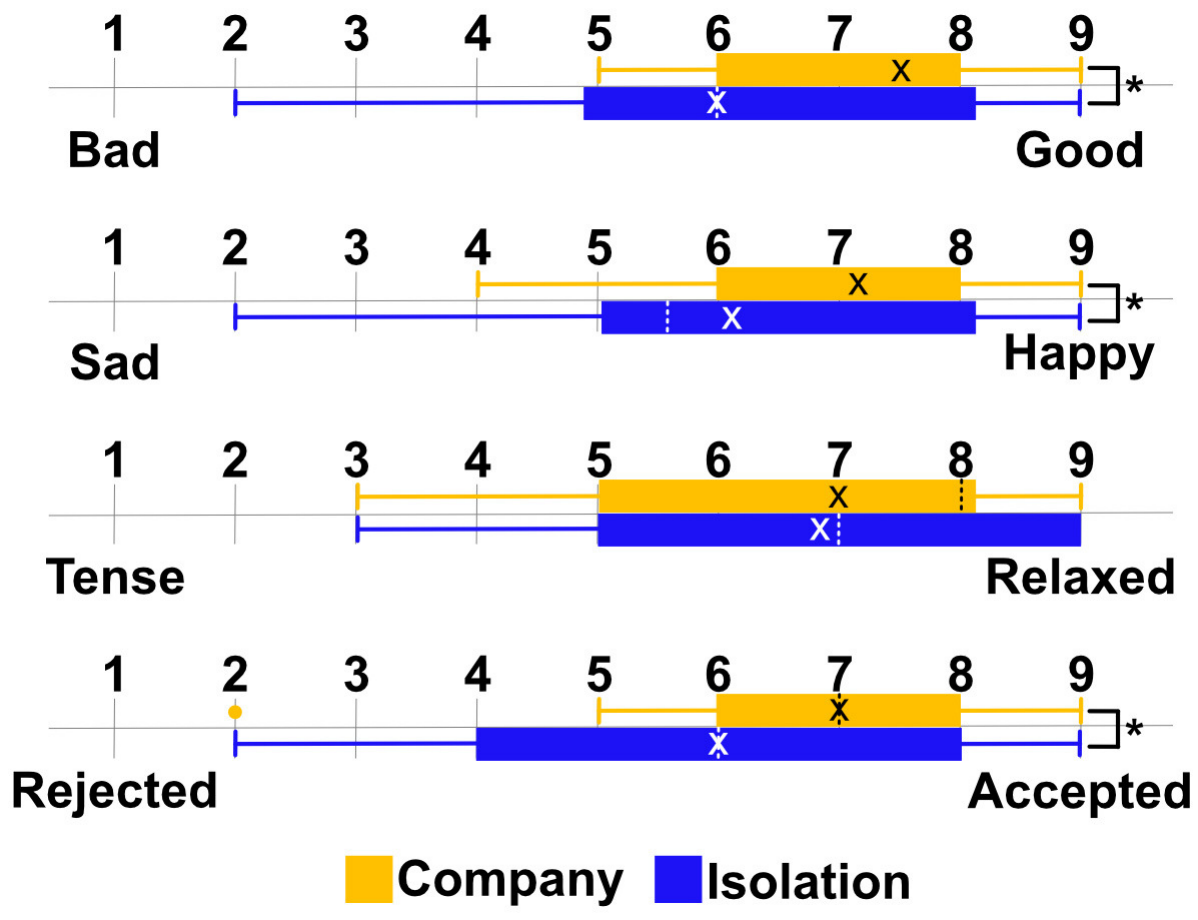
Average of the score for each mood between the company (yellow boxes) and isolation (blue boxes) conditions. The dotted lines indicate the median. *Indicates that the *p*-value was less than 0.013, which is statistically significant after the Bonferroni correction.

Figure 2 indicates the results of the Needs Satisfaction Index (Table 2) between the company and isolation conditions. There were significant differences between the company and isolation conditions in Q1, Q2, Q4, Q5, and Q6 [*p*(21) = 0.006, *p*(21) = 0.007, *p*(21) = 0.003, *p*(21) = 0.001, and *p*(21) = 0.003, respectively, statistically significant as p < 0.008 after the Bonferroni correct]. Furthermore, there were also significant differences in conditions in some scales used to measure mood: Bad–Good, Sad–Happy, Rejected–Accepted [*p*(21) = 0.003, *p*(21) = 0.004, and *p*(21) = 0.010, respectively, statistically significant as *p* < 0.013 after the Bonferroni correction] (Figure 3).

### 3.2 Facial expressions between the company and social isolation conditions

**Figure 4:**
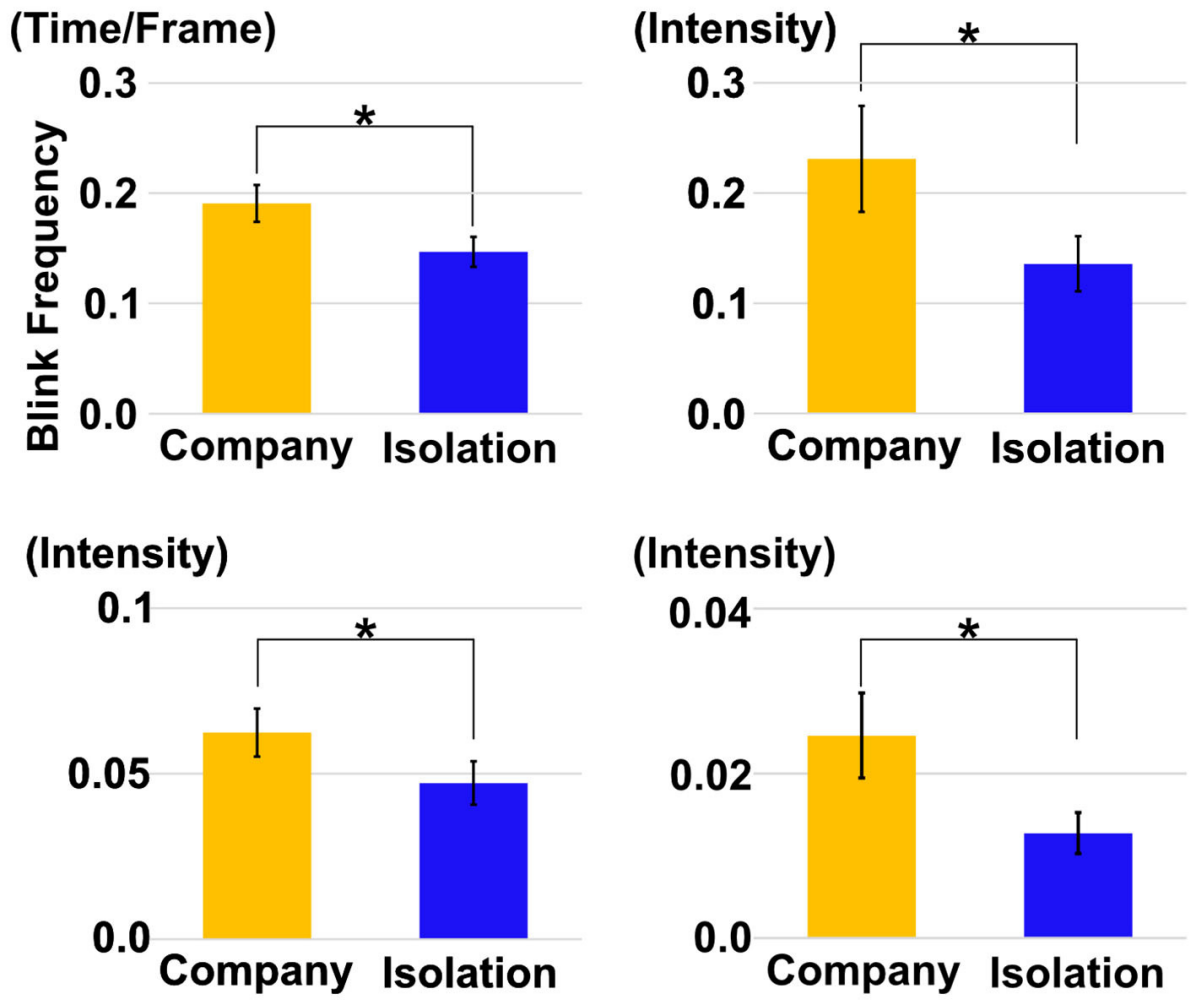
Comparison of the average of AU1, AU2, AU5, and Blink Frequency between company (yellow) and isolation (blue).

The average facial action units around eye muscles, such as AU1, AU2, AU5, and blink frequency, were compared between the isolation and company conditions. The results indicate that all metrics were larger under company conditions than under the isolation condition. Furthermore, paired *t*-tests revealed that these differences were statistically significant, with all p-values being less than 0.05 in AU1, AU2, AU5, and blink frequency [*p*(21) = 0.020, *p*(21) = 0.003, and *p*(21) = 0.020, respectively] (Figure 4).

### 3.3 Eye gaze pattern between the company and isolation conditions

**Figure 5:**
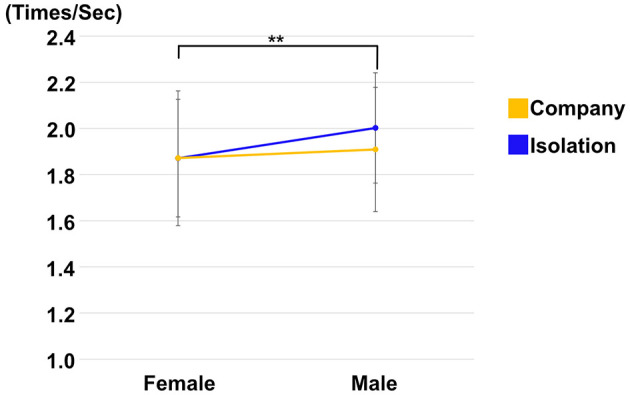
Average of the saccade frequency (Times/Sec) between company (yellow line and circles) and isolation (blue line and circles) conditions. Error bars indicate 95% CI. *Indicates that the *p*-value was less than 0.01.

Gaze calibration failed in one of the participants; thus, the data were excluded from all gaze analyses. In the saccade frequency and fixation duration, a two-way analysis of variance (ANOVA) was performed within experimental conditions (e.g., isolation and company) and gender (e.g., male and female) of the avatars talking was undertaken. In the eye gaze patterns, a three-way ANOVA was conducted within the experimental conditions, gender of the avatars, and talking statements (e.g., the Exclusion and Inclusion states were also considered). There were no significant interactions between the experimental conditions and gender, and among the experimental conditions, gender of avatars, and talking statements in both saccade frequency and fixation duration. On the other hand, the simple effect of the types of gender of avatars was computed, and there was a significant difference between male and female avatars in saccade frequency (F = 13.09, *p*(20) = 0.002, η^2^ = 0.005) (Figure 5).

## 4 Discussion

In this section, the effects of experimental conditions (e.g., company and isolation conditions) on emotions and non-verbal signals are interpreted based on the two aims presented in the Introduction: 1. To develop a task to induce pseudosocial isolation in older adults and 2. To identify non-verbal indicators replacing social isolation in older adults.

### 4.1 Virtual avatar conversation cyberball task inducing pseudo-social isolation

First, with regard to the effect of experimental conditions on feeling social isolation measured using the Needs Satisfaction Index, in the company condition, the scores of all categories were higher than in the social isolation condition in older adults. The results are consistent with the effect of the original cyberball task, in which a participant plays a ball toss game with several computer players through the monitor, in older adults. Hawkley et al. (2011) reported that the original cyberball task induces more social isolation in the isolation condition than in the company condition in older adults. However, participants rated higher scores in the virtual avatar conversation cyberball task than in the original task even in the isolation condition. The results indicate that higher scores for Needs Satisfaction were induced by two factors in this experiment: 1. using a conversation task and 2. using animated avatars. First, a conversation task was used in this experiment instead of the ball toss task. Abdollahi et al. (2022) compared the effect between empathetic (e.g., using facial expressions and response) and non-empathetic (e.g., no facial expression and no response) robots in older adults. There were no significant differences between the empathetic and non-empathetic robots, and both had positive effects on older adults overall (Abdollahi et al., 2022). It can be supposed that communications with robots or virtual avatars generally have a positive impact on the emotions in older adults.

Second, animated virtual avatars were used as interlocutors in this experiment. Fang et al. (2019) investigated that the interface using animated characters with facial expressions induces more positive emotions than other interfaces, such as images, text, and diagrams, in older adults. In this experiment, animated virtual avatars with positive reaction behaviors to participants, such as smiling and nodding, were used; therefore, the experiment had an overall positive effect on the emotions of older adults in both experimental conditions.

### 4.2 Non-verbal signals in social isolation in older adults

Second, with regard to the effect of experimental conditions on non-verbal signals such as facial expressions and gaze patterns measured using a camera and eye tracker, in the social isolation condition, the intensity of the inner/outer brow and upper eyelid raiser and the blink frequency conveying negative emotions such as sadness, anger, or disgust (Sato et al., 2019), are lower than in the company condition. On the other hand, other scientific research highlighted that the inner brow raiser is one of the components of the facial expression that conveys sympathy in Western individuals (Keltner et al., 2019).

With regard to these results, two interpretations are proposed. First, the components of facial expressions conveying each emotion in older adults differ from those in younger adults in the Japanese cohort. Supporting this assumption, Fölster et al. (2014) summarized the difference between older and younger adults in facial expressions using a range of research articles that reported the facial expressions of Western individuals. Age-related changes in flexibility and controllability of muscle tissue may make intentional facial emotion displays less successful with age and unintentional blended emotion displays more likely to occur (Ebner et al., 2011). Second, in the company conditions, older adults felt more empathy toward the avatars than in the isolation conditions because the avatars asked them more questions and engaged them in conversation. Therefore, the intensity of the inner brow raiser expressing empathy was greater in the company condition than in the isolation condition. In future work, to detect detailed emotional information by facial expressions in older adults, a database of facial expressions in Japanese older adults should be developed.

### 4.3 Conclusions

The isolation condition in the virtual avatar communication task based on the cyberball task can successfully elicit social isolation in older adults. Furthermore, social isolation can be detected in older adults by the intensity of the inner/outer brow, upper eyelid raiser, and the blink frequency. On the other hand, the company condition in the avatar communication system can induce empathy in older adults and thus can be used as a mental support system for older adults, especially those living alone. In future work, to develop avatar communication systems for supporting health-related quality of life in older adults, the correlations between social isolation and cognitive decline/mental issues will be better understood by identifying the effect of social isolation on spontaneous brain activities (e.g., the resting state of brain activities) according to the facial expressions of older adults.

### 4.4 Limitations

There are two limitations to this research: 1. Only older adults participated in this project; 2. only men participated in this project. First, this research focused on detecting older adults' social isolation; therefore, only older adults were recruited as participants. To discuss the differences between younger and older adults, younger adults who are younger than 65. should be recruited as participants. Second, it is currently reported that social isolation is more common among men than among women and increases with age (Röhr et al., 2021); therefore, this research focused on older men's social isolation detection.

## Data Availability

The raw data supporting the conclusions of this article will be made available by the authors, without undue reservation.
